# The Toxicology of Native Fucosylated Glycosaminoglycans and the Safety of Their Depolymerized Products as Anticoagulants

**DOI:** 10.3390/md19090487

**Published:** 2021-08-27

**Authors:** Lisha Lin, Sujuan Li, Na Gao, Weili Wang, Taocui Zhang, Lian Yang, Xingzhi Yang, Dan Luo, Xu Ji, Jinhua Zhao

**Affiliations:** 1 State Key Laboratory of Phytochemistry and Plant Resources in West China, Kunming Institute of Botany, Chinese Academy of Sciences, Kunming 650201, China; linlisha@mail.kib.ac.cn (L.L.); lisujuan@mail.kib.ac.cn (S.L.); wangweili@mail.kib.ac.cn (W.W.); zhangtaocui@mail.kib.ac.cn (T.Z.); yanglian@mail.kib.ac.cn (L.Y.); yangxingzhi@mail.kib.ac.cn (X.Y.); 2 College of Life Sciences, University of Chinese Academy of Sciences, Beijing 100049, China; 3 School of Pharmaceutical Sciences, South-Central University for Nationalities, Wuhan 430074, China; gn2008.happy@163.com; 4 College of Traditional Chinese Medicine, Yunnan University of Chinese Medicine, Kunming 650201, China; luodan@mail.kib.ac.cn; 5 School of Chemical Science and Technology, Yunnan University, Kunming 650201, China

**Keywords:** fucosylated glycosaminoglycan, anticoagulant, platelet aggregation, contact activation, hypotension, pulmonary embolism

## Abstract

Fucosylated glycosaminoglycan (FG) from sea cucumber is a potent anticoagulant by inhibiting intrinsic coagulation tenase (iXase). However, high-molecular-weight FGs can activate platelets and plasma contact system, and induce hypotension in rats, which limits its application. Herein, we found that FG from *T. ananas* (TaFG) and FG from *H. fuscopunctata* (HfFG) at 4.0 mg/kg (i.v.) could cause significant cardiovascular and respiratory dysfunction in rats, even lethality, while their depolymerized products had no obvious side effects. After injection, native FG increased rat plasma kallikrein activity and levels of the vasoactive peptide bradykinin (BK), consistent with their contact activation activity, which was assumed to be the cause of hypotension in rats. However, the hemodynamic effects of native FG cannot be prevented by the BK receptor antagonist. Further study showed that native FG induced in vivo procoagulation, thrombocytopenia, and pulmonary embolism. Additionally, its lethal effect could be prevented by anticoagulant combined with antiplatelet drugs. In summary, the acute toxicity of native FG is mainly ascribed to pulmonary microvessel embolism due to platelet aggregation and contact activation-mediated coagulation, while depolymerized FG is a safe anticoagulant candidate by selectively targeting iXase.

## 1. Introduction

Fucosylated glycosaminoglycan (FG) is a sulfated polysaccharide isolated from sea cucumber (*Echinodermata*, *Holothuroidea*), a nutritious food widely consumed in Asia. FG has a unique chemical structure comprised of the backbone of mammal chondroitin sulfate and side chains of sulfated fucoses [1]. Previous study showed that it’s a potent serpin-independent anticoagulant by targeting intrinsic coagulation factor Xase (iXase, FIXa-FVIIIa complex), the last and rate-limiting enzyme in the intrinsic coagulation pathway [2,3,4]. This novel anticoagulant target justifies FG to be a lead compound for safe and effective antithrombosis, the detailed information can be found in a recent review published in Blood Reviews [5]. After depolymerization, the obtained low-molecular-weight FGs (Mw 6~12 kDa) are a selective iXase inhibitor with potent anticoagulant and antithrombotic activities, without obvious bleeding risk [6,7]. Particularly, dHG-5 (Mw 5 kDa), depolymerized from the natural FG from *Holothuria Fuscopunctata* (HfFG) by β-elimination, is an anticoagulant candidate showing superior pharmacological activity compared to enoxaparin in preclinical studies [8,9,10]. Moreover, the clinical trial application of dHG-5 is recently approved by the Food and Drug Administration (IND 153953).

However, native FGs with high molecular weights (Mw > 50 kDa) have multiple pharmacological targets. Among others, they also have the contradictory activities of plasma contact activation (FXII activation) and platelet aggregation [11,12]. These contrary activities may offset their anticoagulant potency, and the lack of selectivity may cause undesired and severe off-target side effects. For instance, a natural FG from *Thelenota ananas* (TaFG) exhibited weaker in vivo antithrombotic activity than its depolymerized product in a stasis-induced venous thrombosis rat model [7]. Native FGs from *Ludwigothurea grisea*, *Isostichopus badionotus*, and *Pearsonothuria graeffei* could cause obvious hypotension and reduced heart rate in rats after intravenous injection [13,14]. However, the underlying mechanism is not clear, and how the undesired activities of FG are related to its in vivo toxicology remains to be studied.

In 2008, heparin contaminated with oversulfated chondroitin sulfate (OSCS) was found to cause an adverse reaction of hypotension in the clinic [15,16,17]. Investigation showed that OSCS induced severe anaphylactoid reactions by activating the plasma contact system, which led to the production of vasoactive bradykinin (BK) and anaphylatoxins (C3a, C5a) [17]. The plasma contact system contains the coagulation factor XII (FXII) and prekallikrein reciprocal activation loop. Contact activation can result in coagulation and inflammation, mediated by coagulation factor XI activation and the kallikrein-kinin pathway, respectively [18]. The contact system can be activated by negatively charged macromolecules such as sulfated polysaccharide [19,20]. Both OSCS and native FG are sulfated polysaccharide and both can potently enhance plasma contact activation; thus, it is assumed that the native FG has the same mechanism as OSCS in inducing hypotension in rats.

In this work, the in vivo toxicology of TaFG, HfFG, and their depolymerized products dTaFG13 (13 kDa) and dHG-5 were studied. Our results reveal that the systemic treatment of native FGs causes severe acute toxicity in rats and the main toxic mechanism was clarified. dTaFG13 had only a very weak effect on rat blood pressure and the anticoagulant candidate dHG-5 showed no side effect in all the assays. Although native FGs have various bioactivities such as antithrombosis, anti-inflammation, anticancer, antivirus, and pro-angiogenesis [1], according to these results, the application of any of their pharmacological activity should be approached cautiously. By contrast, the safety of low-molecular-weight FG as a promising novel anticoagulant is further demonstrated.

## 2. Results and Discussion

### 2.1. In Vivo Effects of FGs in Rats

**Figure 1:**
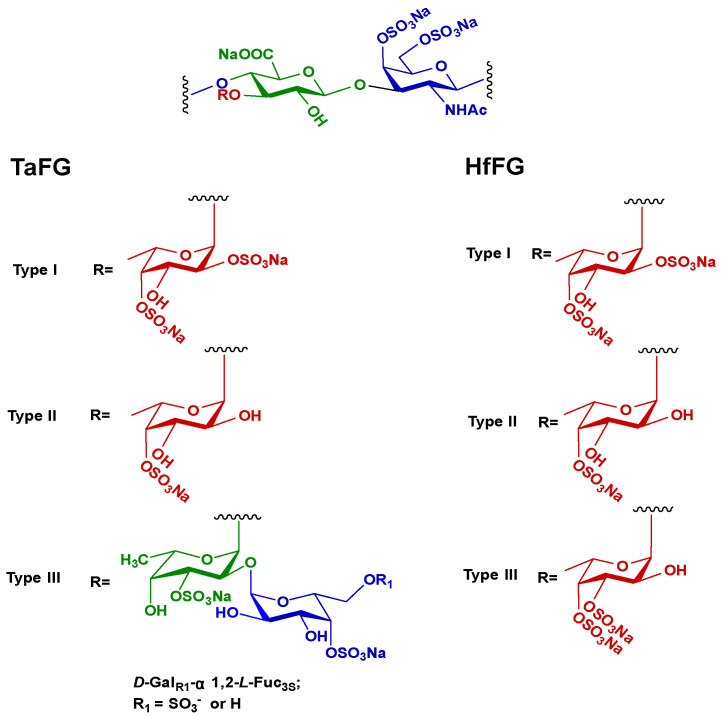
The chemical structures of TaFG and HfFG.

It is reported that high-molecular-weight FG can potently activate the plasma contact system and cause hypotension in rats, similar to OSCS [13,14,16]. Herein, the in vivo effect of TaFG, HfFG, and their depolymerized products were studied. Both TaFG (65.8 kDa) and HfFG (61.1 kDa) have high molecular weights but have different sulfation pattern: 58% of the fucose branches of TaFG are di-sulfated (Fuc2S4S), while 85% of HfFG are di-sulfated (Fuc3S4S) (Figure 1). Unexpectedly, after intravenously injection of TaFG or HfFG (4.0 mg/kg) into the rats, respiration apnea followed by tachypnea could be observed macroscopically, and even death occurred within 5 min. Whereas, the depolymerized FGs caused no obvious macroscopic change. By continuously monitoring some physiological parameters, we found that native FGs caused severe and acute cardiovascular dysfunction and respiratory failure.

#### 2.1.1. Effects of FGs on Rat Blood Pressure

**Figure 2:**
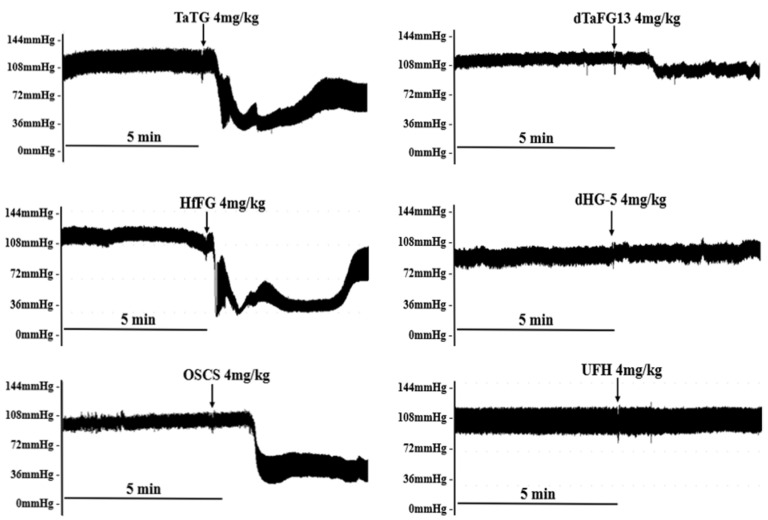
Rat typical arterial pressure response after treatment. Data were recorded by a multi-channel physiological signal-collecting instrument before and after treatment. Arrows indicate the administration of the test compound. Representative graphs are shown, n ≥ 8.

**Figure 3:**
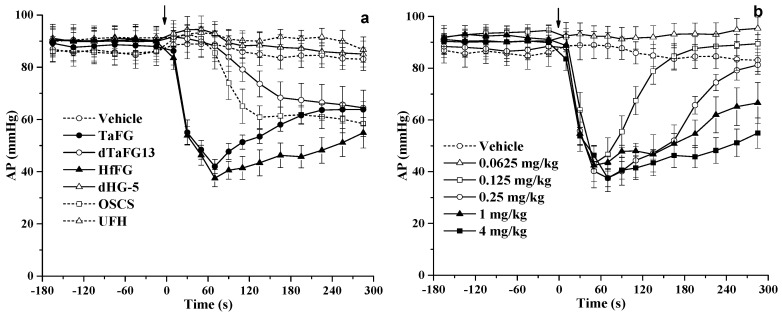
Effects of FG on rat arterial pressure. Effects of different polysaccharides on rat blood pressure (**a**). Effects of different doses of HfFG on rat blood pressure (**b**). After the 10-min adaptation period, rats were treated with a single intravenous bolus of the test compound. The arterial pressure and heart rate were detected for 10 min before and 30 min after treatment. Arrows indicate the administration of the test compound. Mean ± SEM, n ≥ 8. Abbreviation: AP, arterial pressure.

TaFG and HfFG at 4.0 mg/kg injected into the rats caused an immediate drop of arterial pressure (AP) by 55% and 63%, respectively, and their activity was stronger than OSCS, which resulted in hypotension by 35% (Figure 2, Figure 3 and Table S1). The dTaFG13 injection caused mild and a delayed drop of rat blood pressure, while dHG-5 and heparin (UFH) had no obvious effects. The dose–effect relationship of HfFG was also studied (Figure 3 and Table S1). Rats treated with HfFG (0.125~4.0 mg/kg) showed severe hypotension with the similar peak effect at about 1 min, while the recovery time was increased with the dose. HfFG showed the lethal effect at 1 mg/kg and above, and had no obvious effect on blood pressure at 0.0625 mg/kg.

The results are unexpected as such severe side effects of high molecular FG have not been reported by others. Fonseca et al. reported that an FG from sea cucumber *L. grisea* caused a ~40% drop of AP and a slight decrease of heart rate, which was not statistically significant in Wistar rats that received an intravenous dose of 3.5 mg/kg and showed no hypotensive effect when below 3.0 mg/kg [13]. These differences are possibly ascribed to the different types of FG and animal species. Additionally, TaFG and HfFG induced much more acute and severe responses than OSCS, which was reported to induce the human hypotension and anaphylactoid reaction in clinic, mediated by plasma contact system activation [16,17].

#### 2.1.2. Effects of FGs on the Rat Cardiac Function

**Figure 4:**
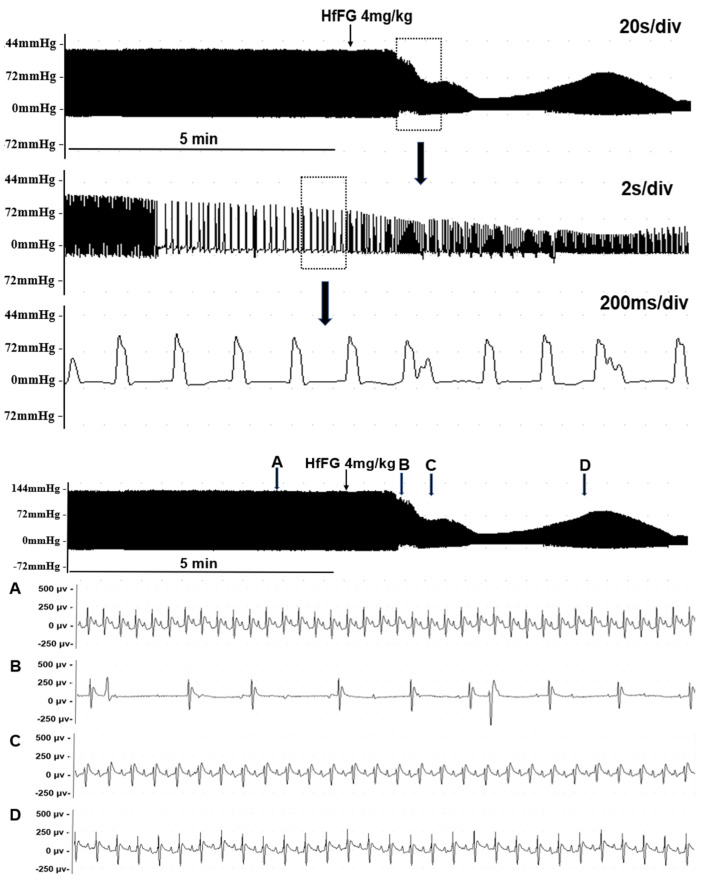
Effect of HfFG (i.v.) on rat left ventricular pressure (**upper**) and electrocardiogram (**below**). Data were recorded by a multi-channel physiological signal-collecting instrument before and after treatment. For the left ventricular pressure, different time scales were shown. For the electrocardiogram, different time points (**A**–**D**) in the left ventricular pressure recording were shown, time scale of (**A**–**D**) was 80 ms/div. Arrows indicate the administration of the test compound. Representative graphs are shown, n ≥ 8.

Then, we investigated the effects of FGs on the rat cardiac function. After TaFG or HfFG (4.0 mg/kg) injection, the rat heart rate (HR), left ventricular pressure (LVP), and maximal rate of rise of ventricular pressure (+dp/dt_max_, indicating the cardiac contractility) were dramatically decreased, reaching to the maximal effect at about 30 s (Table 1 and Figure S1). These changes occurred before the blood pressure drop (described above). A representative graph is shown in Figure 4, TaFG and HfFG reduced LVP by 61% and 68%, respectively, and reduced the heart rate by more than 70% (Table 1). Compared with native FGs, their depolymerized products had no obvious effects on rat cardiac function. OSCS and UFH also had no such effects, to confirm the result, the dose of OSCS was increased up to 8 mg/kg and no obvious change in rat heart rate was observed (data not shown).

#### 2.1.3. Effects of Native FGs on the Rat Respiration

**Figure 5:**
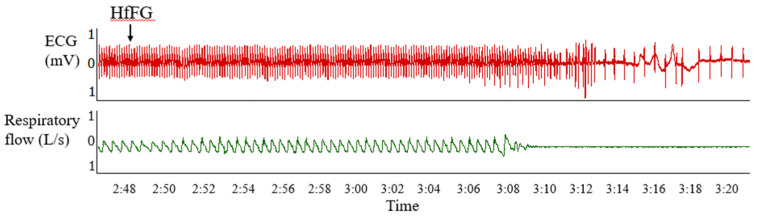
Effects of HfFG (i.v.) on rat ECG and the respiratory wave. Data were recorded by a multi-channel physiological signal-collecting instrument before and after treatment. HfFG was at 4.0 mg/kg and arrows indicate the administration of the test compound. Representative graph is shown and the experiment was repeated three times. Abbreviation: ECG, electrocardiogram.

The effects of TaFG and HfFG on the rat respiratory function were detected simultaneously with the ECG detection. The results showed that after injection of TaFG or HfFG, rats had accelerated respiration, followed immediately by respiratory failure or apnea before the heart rate reduction (Figure 5). This indicates that after native FGs injection, both rat hypotension and cardiac dysfunction occurred secondarily to respiratory dysfunction.

### 2.2. Native FGs Activated the Plasma Contact System in Rats

**Figure 6:**
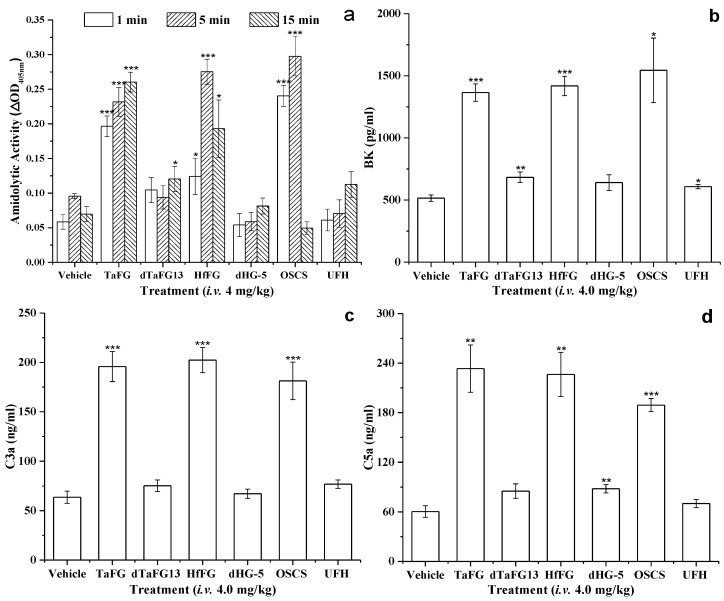
Amidolytic activity (**a**) and levels of BK (**b**), C3a (**c**), and C5a (**d**) in rat plasmas. Amidolytic activity was detected by the chromogenic substrate method and the concentrations of BK, C3a, and C5a were determined by ELISA kits. For each parameter, the value was compared with that of the control (vehicle) group. * *p* ≤ 0.05, ** *p* ≤ 0.01, and *** *p* ≤ 0.001, two-tail *t*-test. Each sample was measured in duplicates and results were expressed as mean ± SEM, n = 3. Abbreviation: BK, bradykinin.

Previously, in vitro assays showed that native FGs exhibit potent activity in plasma contact activation and platelet aggregation [11,12]. As expected, the injection of TaFG and HfFG significantly increased the kallikrein activity of rat plasma, and their activities were comparable to OSCS (Figure 6a). Compared with the control, dTaFG13 slightly enhanced the activation of the contact system in rat plasma, while UFH and dHG-5 caused no significant change. Once the contact system is activated, the reciprocal activation between prekallikrein and FXII produces more kallikrein [18]. Kallikrein can cleave high molecular weight kininogen (HMWK) to generate the vasoactive peptide BK, and mediate the generation of anaphylatoxins C3a and C5a [21,22,23]. These downstream products were detected, plasmas from rats treated with OSCS, TaFG, or HfFG had significantly increased levels of BK, C3a, and C5a, but not those treated with dTaFG13 and dHG-5 (Figure 6b–d).

After production from the plasma contact activation–kallikrein/kinin pathway, BK can cause hypotension effect via kinin B2 receptors (B2R) on endothelial cells [24,25]. Similar to OSCS, native FGs are also negatively charged macromolecules that can potently activate the plasma contact system, leading to the production of vasoactive factor BK. We next investigated the involvement of BK in the toxic effect of native FGs using the B2R antagonist HOE140.

### 2.3. Effects of Inhibitors on the Lethality of Native FGs

HOE140 at 10 μg/kg could completely offset the hypotension induced by 4.0 mg/kg of OSCS or dTaFG13 in rats. However, the hypotension effect of native FGs could not be diminished or abolished by HOE140 (Figure S2). Even rats pretreated with HOE140 at a dose of up to 200 μg/kg showed severe hypotension after TaFG injection. This indicates that the hypotensive effect of native FGs was not mediated by BK, and that their toxic mechanism is different from OSCS. Thus, alternative mechanisms were explored.

Pre-treatment of antiallergic drugs, cholinergic antagonists, and antiasthmatic drugs also cannot prevent the effects of native FGs, whereas we found that pre-treatment of an anticoagulant (heparin or bivalirudin) or antiplatelet (cangrelor) agent can partially mitigate the lethal effect of HfFG (Table 2). In addition, combined the pre-treatment of bivalirudin and cangrelor could completely offset the lethal effect of HfFG. The results indicate that the acute toxic effect of native FGs is mainly caused by thrombosis-mediated embolism, and that their effects of anaphylactoid reaction and cardiovascular failure were secondary pathological changes.

Moreover, the direct effects of native FGs on the vessel vascular tension, rat isolated heart, human myocardial cells, and human Ether-a-go-go Related Gene (hERG) potassium channel were also investigated. The results showed that native FGs exhibited no vasodilative activity and had no direct effects on the functions of the rat isolated heart, myocardial cells (cell proliferation, beat rate, and contractility), and hERG channel (Figures S2–S6)

### 2.4. Effect of FG on Rat Coagulation Function and Platelets

Despite the fact that FG is a potent iXase inhibitor and anticoagulant, high-molecular-weight FGs can also activate FXII and platelets, which may contribute to thrombosis. Previous study showed that, although TaFG had stronger in vitro anticoagulant activity than its depolymerized product, it exhibited weaker in vivo antithrombotic activity in a rat thrombosis model. This may be due to its contradictory activities. Previously, the antithrombotic effect of TaFG was evaluated after subcutaneous injection for 1 h, while the immediate effect of FG after intravenously injection has not been reported.

The anti-iXase IC_50_ values of HfFG and its depolymerized product dHG-5 were 42 ng/mL and 70 ng/mL, respectively [10]. In this work, HfFG and dHG-5 were injected intravenously into rats at 4.0 mg/kg and 6.7 mg/kg, respectively, which were equivalent doses for anti-iXase. Then, the coagulation function was analyzed.

After injection, TaFG and HfFG at 4.0 mg/kg significantly prolonged rat plasma APTT due to their anti-iXase activity, while shortening PT (Table 3). At 15 min, TaFG-treated rats not only had the largest APTT but also had prolonged PT. This indicates that native FG exhibited procoagulant activity in vivo immediately after injection, while coagulopathy occurred later due to the coagulation factor consumption. By comparison, dHG-5 at 6.7 mg/kg prolonged rat plasma APTT and had no effect on PT. According to APTT, TaFG showed stronger anticoagulant activity than HfFG. We also found that TaFG only slightly increased the D-dimer, while HfFG significantly increased the D-dimer level in rat plasmas (Table S2). Combined with the finding of suspected pulmonary embolism in the autopsy of rats treated with native FGs, these results may explain the finding that HfFG (6/6) caused more death than TaFG (2/8).

After injection, TaFG and HfFG at 4.0 mg/kg dramatically decreased the platelet count by 84.6% and 86.4%, respectively, while dHG-5 at 6.7 mg/kg had no such effect (Table 4). The platelet reduction in blood may be due to the platelet aggregation induced by native FGs, which is consistent with their in vitro activity of platelet activation [5].

### 2.5. Native FGs Induced Rat Pulmonary Embolism

**Figure 7:**
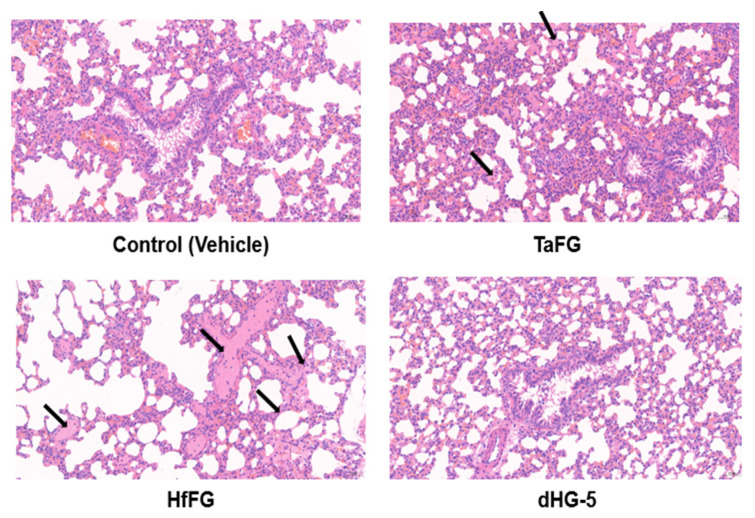
Rat pulmonary microvessel embolism induced by native FGs. Rat were i.v. injected with TaFG (4.0 mg/kg), HfFG (4.0 mg/kg), dHG-5 (6.7 mg/kg), or the vehicle and after 5 min the lung was collected for histopathological assays. Experiments were conducted in triplicates and represented results are shown.

We then further studied the histopathology of rat essential organs including the lung and heart after FG injection. Consistent with their in vivo procoagulant effect, TaFG and HfFG at 4.0 mg/kg caused microvessel embolism and fibrin deposition in rat lungs, while dHG-5 had no effect on lung histology (Figure 7). No microthrombus was found in the heart pathological section in all groups (Table S3). Taken together, the lethal effect of native FG may be mediated by pulmonary embolism due to their activities in the contact system and platelet activation, as confirmed by the remedy effect of pretreated antithrombotic drugs (anticoagulant bivalirudin combined with antiplatelet cangrelor). In addition, after injection of TaFG or HfFG, rats had apnea or respiratory failure prior to cardiovascular dysfunction.

Apart from the activity of procoagulation and inducing the platelet aggregation, the increased production of C3a and C5a by TaFG or HfFG may also contribute to the accumulation and degradation of platelets in the lung, as the involvement of the complement system in lipopolysaccharide-induced anaphylaxis-like shock has been reported [23,26,27,28].

## 3. Conclusions

Previous study reported that FGs from sea cucumber have various bioactivities. However, the results of our work indicate that native FGs (TaFG and HfFG) injected intravenously showed strong and acute toxicity in rats. The toxic effect and practical significance of native FGs should be noted when conducting pharmacological studies, especially those involving systemic treatment.

After the injection into rats, the acute toxic effects of TaFG and HfFG involved a dramatic reduction of blood pressure, cardiac dysfunction, respiratory failure, coagulopathy, and decreased platelet count. Although TaFG and HfFG were similar to OSCS in activating the plasma contact system, they showed much more severe toxic effects and the underlying toxic mechanisms were different from OSCS. Pre-treatment of a BK receptor antagonist completely offset the hypotension induced by OSCS, while it had no effect on that induced by TaFG and HfFG. By applying the prophylactic drugs, we found that anticoagulant combined with an antiplatelet can completely prevent the lethality of TaFG and HfFG, while other inhibitors had no obvious preventive effects. This indicates that thrombosis is an initial key factor in the lethal toxicity of these native FGs. Previously, in vitro assays have shown that native FGs can activate platelets and trigger contact activation–coagulation pathway. In this work, these effects were also detected in the rat blood or plasma after treatment. Additionally, TaFG and HfFG caused disseminated pulmonary embolism as revealed by the histopathological analysis.

In summary, the main mechanism of the acute toxicity of native FG was ascribed to the pro-coagulation and platelet activation, which further led to pulmonary embolism and respiratory failure. The side effect of FG depends on the high molecular weight but not the sulfation pattern. After chemical depolymerization, dTaFG13 (13 kDa) and dHG-5 (5 kDa) had no obvious effects on all the tested physiology parameters, which is consistent with their lack of platelet and contact activation activities in vitro. Therefore, depolymerized FG can be safe and effective anticoagulant candidate by selectively targeting the intrinsic coagulation pathway.

## 4. Materials and Methods

### 4.1. Drugs and Chemicals

The preparation and physicochemical characteristics of FGs with various molecular weight were described in previous studies [6,7,29]. The compound TaFG and HfFG were prepared from *T. ananas* and *H. fuscopunctata*, respectively. Depolymerized dTaFG13 (13 kD) and dHG-5 (5 kD) were prepared from TaFG and HfFG, respectively. OSCS and heparin (UFH, 197 IU/mg) were purchased from the National Institute for the Control of Pharmaceutical and Biological Products (Beijing China). CS31(02) (D-Pro-Phe-Arg-pNa, Kallikrein chromogenic substrate) was obtained from Hyphen BioMed (Neuville-sur-Oise France). HOE140 (B2R antagonist) and the protease inhibitor cocktail were obtained from Sigma-Aldrich. Bivalirudin and cangrelor were obtained from Targetmol. The Rat BK, C3a, and C5a ELISA kits were obtained from Shanghai Guyan Real Co., Ltd. (Shanghai, China). The activated partial thromboplastin time (APTT) and prothrombin time (PT) were obtained from TECO GmbH (Neufahrn, Germany). The Rat D-Dimer (D2D) ELISA Kit was obtained from SAB (Baltimore, MD, USA). All other chemicals were of reagent grade and were obtained commercially.

### 4.2. Animals and Biological Samples

Male Sprague-Dawley rats (body weight of 250~350 g) were obtained from Hunan SJA Laboratory Animal Co. Ltd. (Changsha, China). Rats were housed in animal rooms with controlled temperatures (18–25 °C) and relative humidity (40~60%), and were allowed to eat and drink ad libitum before experiments. These experiments were reviewed and approved by the Animal Ethics Committee of the Kunming Institute of Botany, Chinese Academy of Sciences (SYXK (Dian) K2018-0005).

Rat blood were collected using a polyethylene catheter from the jugular vein before and after 1, 5, and 15 min following the treatment with FG, OSCS, UFH, or the vehicle (normal saline). Nine volumes of blood was anticoagulated with one volume of 1% EDTA (for the blood used for the clotting assays, 3.8% sodium citrate was used instead). Then, rat plasmas were obtained after centrifugation (1000× *g*, 15 min). Plasma samples were stored at −80 °C or assayed immediately. For the plasma using for BK detection, 10% Protease Inhibitor Cocktail was added.

### 4.3. Rat Plasma Contact Activation Analysis

Rat blood (0.3 mL) was collected before and after 1 min, 5 min, and 15 min of each treatment. Rat plasma samples were obtained as described in Section 4.2. After dilution with 4 volumes of TS buffer (50 M Tris-HCl, 150 M NaCl, pH 7.4), 100 µL of rat plasma was added into a microwell plate and incubated at 37.0 °C for 60 s. Then, the amidolytic activity of the plasma was assessed by adding 30 µL of 1 mM CS31(02) (chromogenic substrate of kellikrein). The optical density at 405 nM (OD_405nm_) was recorded at 37.0 °C for 20 min using the Bio-Tek Microplate Reader (ELx 808, Winooski, VT, USA) and change of OD_405nm_ was calculated and expressed as ΔOD_405nm_.

### 4.4. Rat Plasma Levels of BK, C3a, and C5a Determination

The concentration of BK, C3a, and C5a in plasma samples collected from rats after 1 min of treatment with FG, OSCS, or normal saline were assayed by means of ELISA (enzyme-linked immunosorbent assay), as specified in the manufacturer’s instructions.

### 4.5. Rat Blood Pressure and Cardiac Function Detection

After rats were anesthetized and fixed, the left jugular vein and right common carotid artery were separated from the tissue and intubated. The inserted catheters were filled with heparinized (125 U/mL) normal saline, the free end of the vein catheter was connected to an injection syringe for drug injection/blood collection, and the artery catheter was connected to a pressure amplifier for blood/ventricular pressure detection. In some experiments, rat electrocardiograms (ECG) were recorded simultaneously by electrodes. Data were acquired by a multi-channel physiological signal-collecting instrument (RM6240, Chengdu, China) with a continuous recording pattern. After a stable period, the compound (1 mL/kg) was intravenously injected.

The systolic blood pressure (SBP), diastolic blood pressure (SDP), and the left ventricular pressure (LVP), were recorded for 10 min before and 30 min after treatment, and the heart rate (HR), mean arterial pressure (AP), pulse pressure (SBP-DBP), and maximal rates of rise of ventricular pressure (+dP/dt_max_) were calculated. The change of AP (ΔAP), HR (ΔHR), or LVP (ΔLVP) were calculated by subtracting the average value of the peak effect after treatment from the average value before treatment (if there was no obvious effect, the value of around 1 min post-dose was used). The change rate, such as ΔAP (%), was calculated as: ΔAP/AP(pre-dose) ∗ 100%.

### 4.6. Rat Respiratory Function Detection

After rats were anesthetized and fixed, the trachea was exposed and intubated. The free end of the catheter was connected to a respiratory flow head which linked to PowerLab (AD instruments, New South Wales, Australia). The respiratory wave was recorded 3 min before and 30 min after treatment. Rat electrocardiograms (ECG) were recorded simultaneously by electrodes connecting to PowerLab.

### 4.7. Effect of the B2R Antagonist on Rat Hypotension

Anesthetized rats were intravenously injected with 40 μg/kg of HOE140, a highly specific B2R antagonist, 3 min prior to the treatment with FGs or OSCS at 4.0 mg/kg. The record of the rat blood pressure and heart rate were as described in Section 4.5. The dosage of HOE140 was in reference to the literature which stated that 10 μg/ml of HOE140 could fully inhibit the hypotensive effect of 30 μg/kg of BK [16] and validated our pre-experiment.

### 4.8. Effects of Antithrombotic Drugs on the Toxicity of Native FG

Anesthetized rats were intravenously injected with 1 mL/kg of heparin, bivalirudin, cangrelor bivalirudin combined with cangrelor, or normal saline (control). After 3 min, rats were injected with 4.0 mg/kg of HfFG. The responses of rats after treatment were observed for 1 h and the death time within 1 h was recorded.

### 4.9. Rat Coagulation Function and Platelet Count Analysis

Rats were anesthetized and the left jugular vein was separated and intubated for drug injection/blood collection. The inserted catheter was filled with normal saline. Rat blood was collected before and after FG treatment, and plasma was obtained as described in Section 4.2. The plasma APTT and PT were detected by a coagulometer (TECO MC-4000, Neufahrn, Germany) using APTT and PT reagents. The plasma D-dimer level was assayed by means of ELISA as specified in the manufacturer’s instructions.

### 4.10. Rat Histopathologic Analysis after Treatment

Anesthetized rats were intravenously injected with FG or saline and after 5 min of treatment, the lung and heart were collected and immersed in 4% paraformaldehyde. After 24 h, the tissue samples were embedded in paraffin, cut into 4 μm thin sections, and stained with hematoxylin-eosine. The samples were examined using the Nikon Eclipse Ci-L microscope and analyzed by 3DHISTECH software (Budapest, Hungary).

### 4.11. Statistical Analysis

All data are given as the mean ± SEM. The data were analyzed using Student’s *t*-test (two-tail) compared with the control group or pretreated values. P values less than 0.05 were considered statistically significant (* *p* < 0.05, ** *p* < 0.01, or *** *p* < 0.001).

## Figures and Tables

**Table 1:** Effects of FGs on the rat cardiac function (mean ± SEM, n ≥ 8).

Treatment (mg/kg)	mLVP_0_ (mmHg)	mLVP_1_ (mmHg)	(+dP/dt_max_)_0_ (10^3^ mmHg/s)	(+dP/dt_max_)_1_ (10^3^ mmHg/s)	ΔHR (%)
Vehicle	47.2 ± 3.3	48.7 ± 2.3	4.8 ± 0.3	4.9 ± 0.3	−0.7 ± 1.4
TaFG (4.0)	47.4 ± 2.3	18.3 ± 3.2 ***	3.8 ± 0.4	1.4 ± 0.2 ***	−71.0 ± 5.8
dTaFG13 (4.0)	39.5 ± 2.5	44.9 ± 3.0	4.3 ± 0.6	4.5 ± 0.5	−3.9 ± 2.1
HfFG (4.0)	47.8 ± 2.3	15.4 ± 2.5 ***	3.8 ± 0.4	1.4 ± 0.2 ***	−72.9 ± 5.0
dHG-5 (4.0)	42.4 ± 2.2	42.5 ± 2.4	4.0 ± 0.4	4.2 ± 0.5	3.8 ± 2.3
OSCS (4.0)	46.6 ± 3.2	35.0 ± 2.3 *	3.3 ± 0.3	3.2 ± 0.4	14.1 ± 7.1
UFH (4.0)	43.5 ± 2.4	47.3 ± 2.3	3.7 ± 0.4	4.3 ± 0.5	5.3 ± 7.4
HfFG (1.0)	46.3 ± 3.2	16.5 ± 1.8 ***	3.7 ± 0.4	1.2 ± 0.1 ***	−77.7 ± 2.7
HfFG (0.25)	45.1 ± 2.4	17.1 ± 2.1 ***	3.1 ± 0.4	1.4 ± 0.2 **	−63.7 ± 6.6

*p ≤ 0.05, ** *p* ≤ 0.01, and *** *p* ≤ 0.001 vs. pretreatment, two-tail t-test. The subscript 0 and 1 indicate the value before and after treatment, respectively. Abbreviations: mLVP, mean left ventricular pressure; +dP/dt_max,_ the maximal rates of rise of ventricular pressure; and ΔHR, the change of heart rate.

**Table 2:** Effects of inhibitors on rat mortality induced by FGs (i.v.).

Treatment	Pre-Treatment	Mortality/Total ^a^	Average Death Time Post-Dose
HfFG (4.0 mg/kg)	Saline (1 mL/kg)	4/4	5 min
	Heparin (5.0 mg/kg)	1/3	-
	Bivalirudin (2.5 mg/kg)	3/3	15 min
	Cangrelor (0.5 mg/kg)	1/3	-
	Bivalirudin (2.5 mg/kg), Cangrelor (0.5 mg/kg)	0/5	-

^a^ The ratio of death numbers to total rats within 60 min after treatment.

**Table 3:** Effects of FGs (i.v.) on the rat coagulation function (mean ± SEM).

Treatment	Dose (i.v.)	Animals	Mortality ^a^	Blood Collection Time ^b^	APTT (s) ^c,d^	PT (s) ^d^
TaFG	4.0 mg/kg	8	2	−1 min	15.5 ± 0.3	23.4 ± 0.6
				1 min	295.2 ± 3.8 ***	15.8 ± 0.7 ***
				5 min	208.0 ± 39.9 *	16.7 ± 0.4 ***
				15 min	300.0 ± 0.0 ***	36.5 ± 3.6 **
HfFG	4.0 mg/kg	6	6	−1 min	16.1 ± 0.6	25.1 ± 1.0
				1 min	227.9 ± 10.3 ***	16.2 ± 0.7 ***
				5 min	116.0 ± 6.2 ***	16.1 ± 1.1 ***
				15 min	-	-
dHG-5	6.7 mg/kg	5	0	−1 min	16.2 ± 0.7	26.9 ± 1.1
				1 min	196.8 ± 27.3 **	27.2 ± 1.2
				5 min	73.5 ± 7.7 **	27.4 ± 0.8
				15 min	42.4 ± 4.4 **	26.8 ± 1.3

^a^ The death numbers within 15 min after drug treatment. ^b^ Animals were treated with FG at 0 min (−1 min was before treatment). ^c^ The APTT beyond detection limit (>300 s) was recordedas 300 s. ^d^ Compared with the control (before treatment): * *p* < 0.05, ** *p* < 0.01, and *** *p* < 0.001 (two-tail *t*-test).

**Table 4:** Effects of FGs (i.v.) on the rat platelet count (mean ± SEM, n = 3).

Treatment	Dose	Platelet Count (10^9^/L)		Platelet Count Reduction (%)
		Pretreatment	1 min Post-Dose	
Control	-	363 ± 19	337 ± 8	7.1
TaFG	4.0 mg/kg	331 ± 25	51 ± 9 ***	84.6
HfFG	4.0 mg/kg	250 ± 32	34 ± 8 **	86.4
dHG-5	6.7 mg/kg	327 ± 37	354 ± 28	−8.3

** *p* < 0.01, and *** *p* < 0.001 vs. pretreatment (two-tail *t*-test).

## Data Availability

All data is contained within this article and the Supplementary Materials.

## Supplementary Materials

The following are available online at https://www.mdpi.com/article/10.3390/md19090487/s1, Table S1: Effects of FGs on rat blood pressure (mean ± SEM); Table S2: Effects of FGs on rat plasma D-dimer (mean ± SEM, n = 3); Table S3: Pathological scores of rat heart and lung after FGs (i.v.) treatment; Figure S1: Effects of FGs on rat left ventricular pressure and the heart rate; Figure S2: Effects of FGs on rat AP and HR in rats with or without HOE140 pre-treatment; Figure S3: Effects of TaFG and HfFG on the pre-contracting rat aortic artery; Figure S4: Effects of HfFG on the function of the rat isolated heart; Figure S5: Effects of native FGs on hERG k^+^ current; and Figure S6: Effects of HfFG on the function of human myocardial cells.
