# Supplementary material for: The Toxicology of Native Fucosylated Glycosaminoglycans and the Safety of Their Depolymerized Products as Anticoagulants
Source: Mar Drugs. 2021 Aug 27;19(9):487. doi: 10.3390/md19090487 (PMC8467514; doi:10.3390/md19090487)
Supplement: Supplementary file 1 [file marinedrugs-19-00487-s001.zip › marinedrugs-1343035-supplementary.pdf]

Supporting Information:

The toxicology of native fucosylated glycosaminoglycans and the safety of their depolymerized products as anticoagulants

Lisha Lin[1,2], Sujuan Li[1,2], Na Gao[3], Weili Wang[1,2], Taocui Zhang[1,2], Lian Yang[1], Xingzhi Yang[1], Dan Luo[4], Xu Ji[5*], Jinhua Zhao[3*]

1 State Key Laboratory of Phytochemistry and Plant Resources in West China, Kunming Institute of Botany, Chinese Academy of Sciences, Kunming 650201, China; linlisha@mail.kib.ac.cn (L.L.); lisujuan@mail.kib.ac.cn (S.L.); wangweili@mail.kib.ac.cn (W.W.); zhangtaocui@mail.kib.ac.cn (T.Z.); yanglian@mail.kib.ac.cn (L.Y.); yangxingzhi@mail.kib.ac.cn (X.Y.)

2 University of Chinese Academy of Sciences, College of Life Sciences, Beijing 100049, China;

3 School of Pharmaceutical Sciences, South-Central University for Nationalities, Wuhan, 430074, China; gn2008.happy@163.com (N.G.); zhao.jinhua@yahoo.com (J.Z.)

4 College of Traditional Chinese Medicine, Yunnan University of Chinese Medicine, Kunming, 650201, China; luodan@mail.kib.ac.cn (D.L.)

5 School of Chemical Science and Technology, Yunnan University, Kunming, 650201, China; jixu@ynu.edu.cn (X.J.)

* Correspondence: jixu@ynu.edu.cn (X.J.); zhao.jinhua@yahoo.com (J.Z.)

Contents：

Table S1. Effects of FGs on rat blood pressure (Mean ± SEM)
Table S2 Effects of FGs on rat plasma D-dimer (Mean ± SEM, n = 3)
Table S3 Pathological scores of rat heart and lung after FGs (*i.v.*) treatment
Fig. S1 Effects of FGs on rat left ventricular pressure and heart rate
Fig. S2 Effects of FGs on rat AP and HR in rats with or without HOE140 pre-treatment
Fig. S3 Effects of TaFG and HfFG on pre-contracting rat aortic artery
Fig. S4 Effects of HfFG on the function of rat isolated heart
Fig. S5 Effects of native FGs on hERG k^+ current
Fig. S6 Effects of HfFG on the function of human myocardial cells

Table S1. Effects of FGs on rat blood pressure (Mean ± SEM)

Treatment	Doses (mg/kg)	Number of animal	Number of death	ΔAP (mmHg)[a,b]	ΔAP (%)[b]	Δ(SBP-DBP) (mmHg)[a,c]
Vehicle	-	8	0	0.1 ± 2.3	-0.2 ± 2.8	0.4 ± 0.6
TaFG	4.0	11	3	-48.4 ± 3.0***	-55.1 ± 2.5	-8.2 ± 2.7*
dTaFG13	4.0	8	0	-19.8 ± 4.3*	-22.0 ± 4.4	4.2 ± 1.0
HfFG	4.0	13	5	-59.5 ± 4.5***	-63.3 ± 3.3	-10.1 ± 2.0**
dHG-5	4.0	9	0	5.5 ± 2.2	6.9 ± 3.0	-2.3 ± 1.2
OSCS	4.0	8	0	-31.1 ± 4.6***	-35.1 ± 4.5	8.8 ± 1.7*
UFH	4.0	9	0	0.5 ± 1.8	1.0 ± 2.4	0.3 ± 0.6
HfFG	1.0	12	3	-58.4 ± 3.4***	-63.0 ± 2.8	2.1 ± 3.2
HfFG	0.25	8	0	-54.8 ± 5.2***	-62.2 ± 5.8	-3.2 ± 5.1
HfFG	0.125	8	0	-54.5 ± 6.7***	-57.9 ± 7.1	1.9 ± 5.3
HfFG	0.0625	8	0	1.5 ± 3.8	4.2 ± 6.1	-1.0 ± 1.0

[a]For each parameter, the value of peak effect was compared with the predose basal value of the same group, *$P \leq 0.05$, **$P \leq 0.01$ and ***$P \leq 0.001$, two-tail tTest.

[b]ΔAP, the change of arterial pressure.

[b]ΔSBP-DBP, the change of pulse pressure (systolic blood pressure-diastolic blood pressure).

Table S2 Effects of FGs on rat plasma D-dimer (Mean ± SEM, n = 3)

Treatment	Dose	Blood collected time[b]	D-dimer (ng/mL)[a]
TaFG	4.0 mg/kg	-1 min predose	7.5 ± 2.6
		1 min postdose	12.6 ± 3.4
		5 min postdose	17.6 ± 6.6
		15 min postdose	10.7 ± 4.5
HfFG	4.0 mg/kg	-1 min predose	11.1 ± 3.5
		1 min postdose	32.9 ± 5.3*
		5 min postdose	48.6 ± 16.2
dHG-5	6.7 mg/kg	-1 min predose	10.4 ± 5.8
		1 min postdose	19.4 ± 3.3
		5 min postdose	2.9 ± 0.0
		15 min postdose	13.3 ± 10.2

[d] Compare with control (before treatment): **P* < 0.05 (two-tail t-test).

Table S3 Pathological scores of rat heart and lung after FGs (*i.v.*) treatment

Treatment	Animal	Pulmonary microvessel embolism	Heart bleeding	Myocardial infarction
Vehicle (saline)	1-1	0	0	0
	1-2	0	0	0
	1-3	0	0	0
TaFG (4.0 mg/kg)	2-1	3	0	0
	2-2	2	0	0
	2-3	2	1	0
HfFG (4.0 mg/kg)	3-1	3	0	0
	3-2	3	2	0
	3-3	2	0	0
dHG-5 (6.7 mg/kg)	4-1	0	0	0
	4-2	0	0	0
	4-3	0	0	0

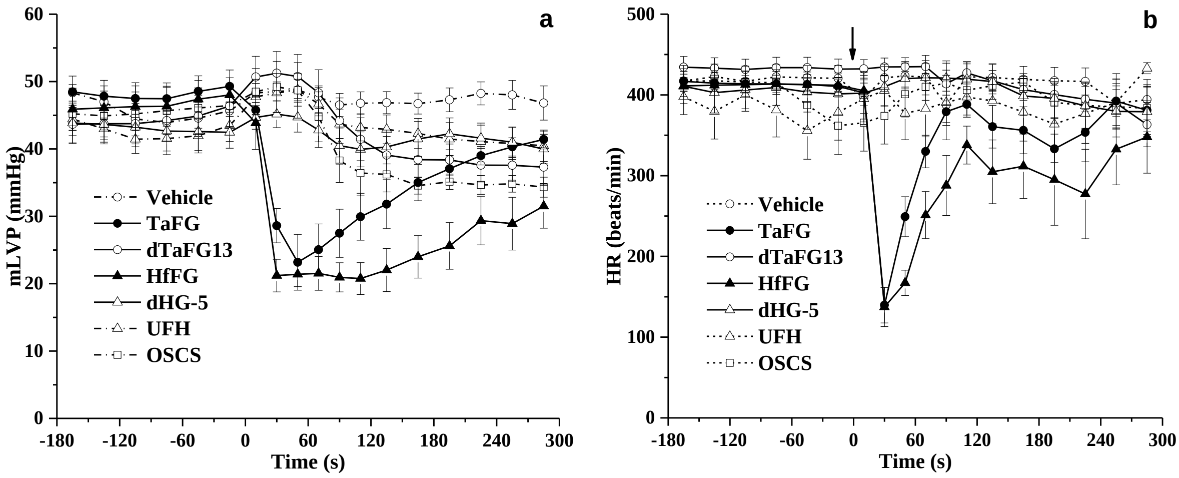

Fig. S1 Effects of FGs on rat left ventricular pressure and heart rate

Arterial catheter connected to a pressure transducer was inserted into left ventricle, after an adaptation period of 10 min, rats received a single intravenous bolus (4.0 mg/kg) of TaFG, dTaFG13, HfFG, dHG-5, OSCS, UFH, or vehicle, LVP or HR was recorded before and after drug administration. Arrows indicate the administration time, Mean ± SEM, n ≥ 8. mLVP, mean left ventricular pressure; HR, heart rate.

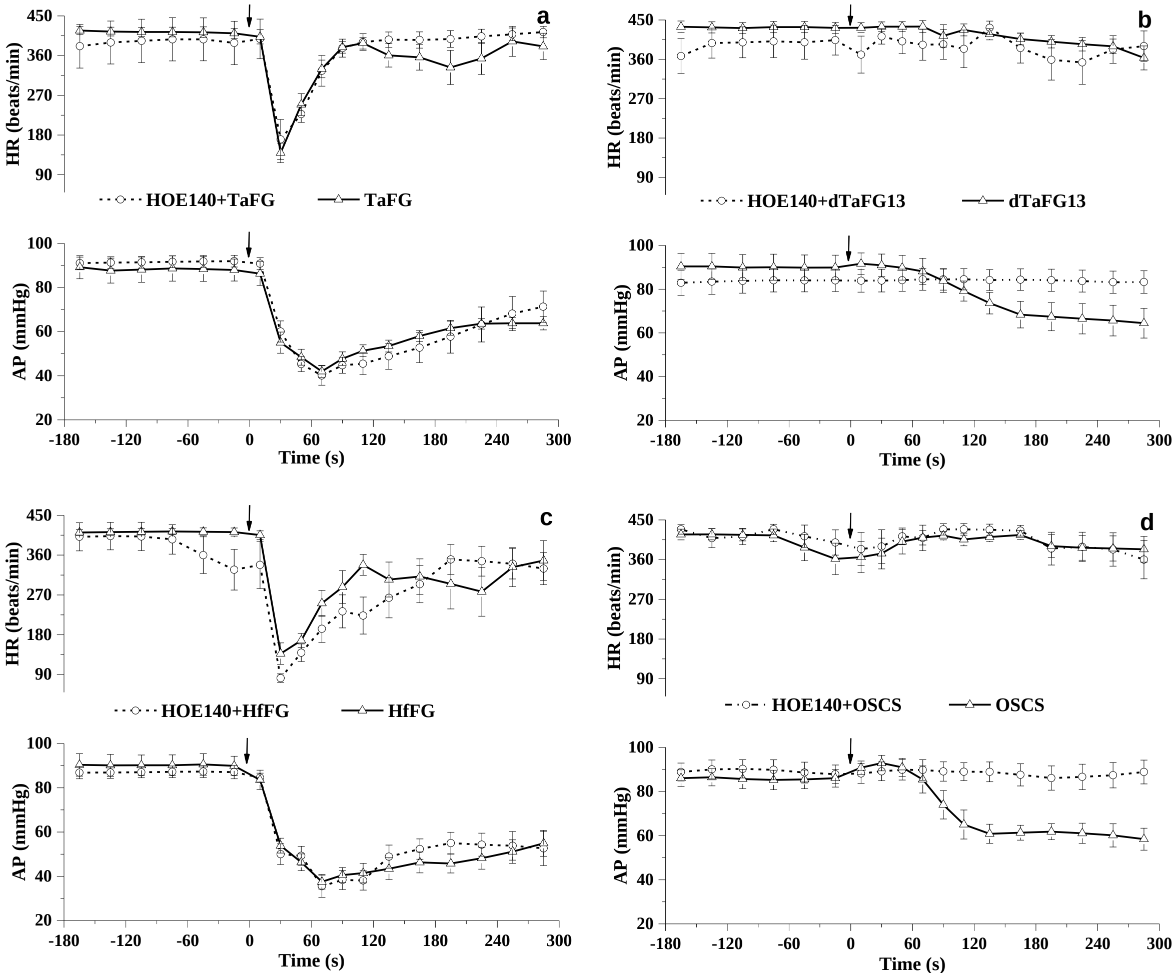

Fig. S2 Effects of FGs on rat AP and HR in rats with or without HOE140 pre-treatment

After 10 min adaptation period, rats received 4.0 mg/kg TaFG(a), dTaFG13 (b), HfFG (c) or OSCS (d), with or without pretreatment of 10 μg/kg HOE140. Arterial pressure and heart rate were recorded as previously described. Arrows indicate the administration of test compound. Mean ± SEM, n = 6~8.

A

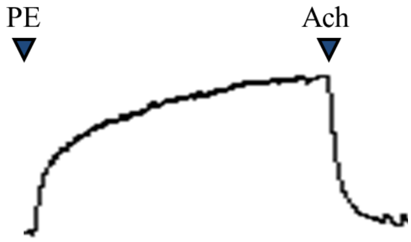

B

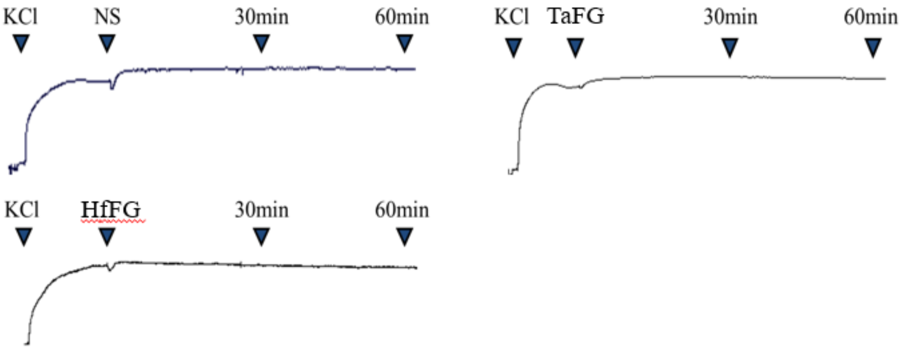

Fig. S3 Effects of TaFG and HfFG on pre-contracting rat aortic artery

(A) The vasodilative effect of acetylcholine on phenylephrine pre-contracting vessel (to validate the endothelium integrity); (B) Effects of compound on the tension of KCl pre-contracting vessel, in control group, same volume of normal saline (NS) was added.

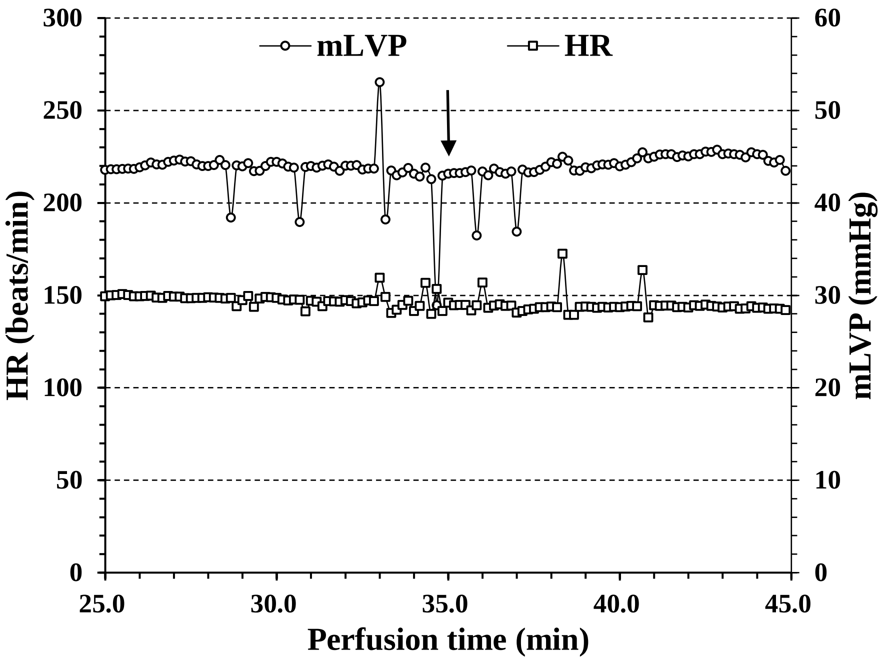

Fig. S4 Effects of HfFG on the function of rat isolated heart

The arrow indicated the drug-added time, mLVP: mean left ventricle pressure, HR: heart rate; experiment was repeated twice, and the average data were expressed.

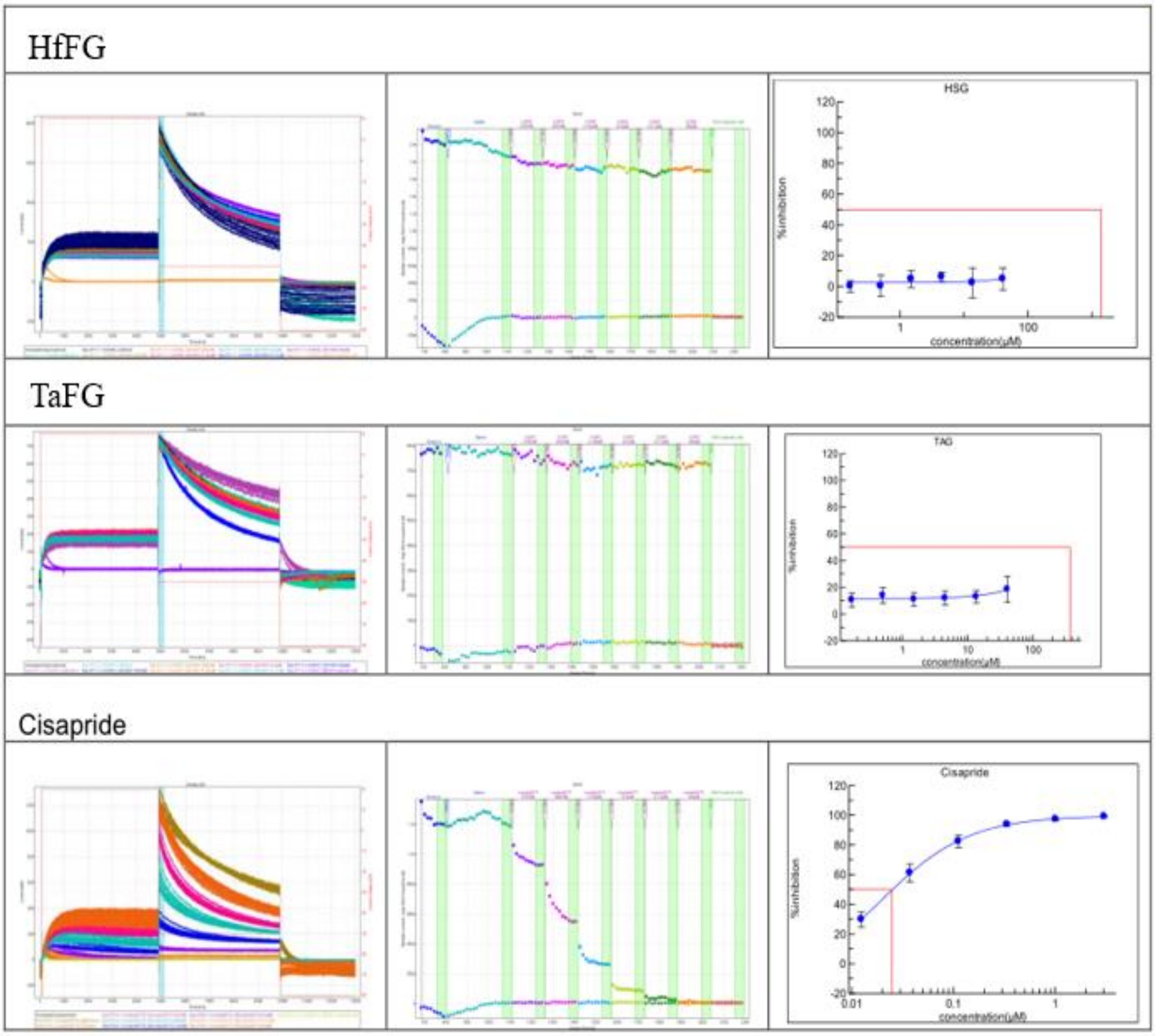

Fig. S5 Effects of native FGs on hERG k+ current

(Left) Effects of compound on hERG channel; (Middle) time-K+ current figure; and (Right) dose-effect figure. Cisapride is a positive control. Mean ± SEM, n ≥ 3.

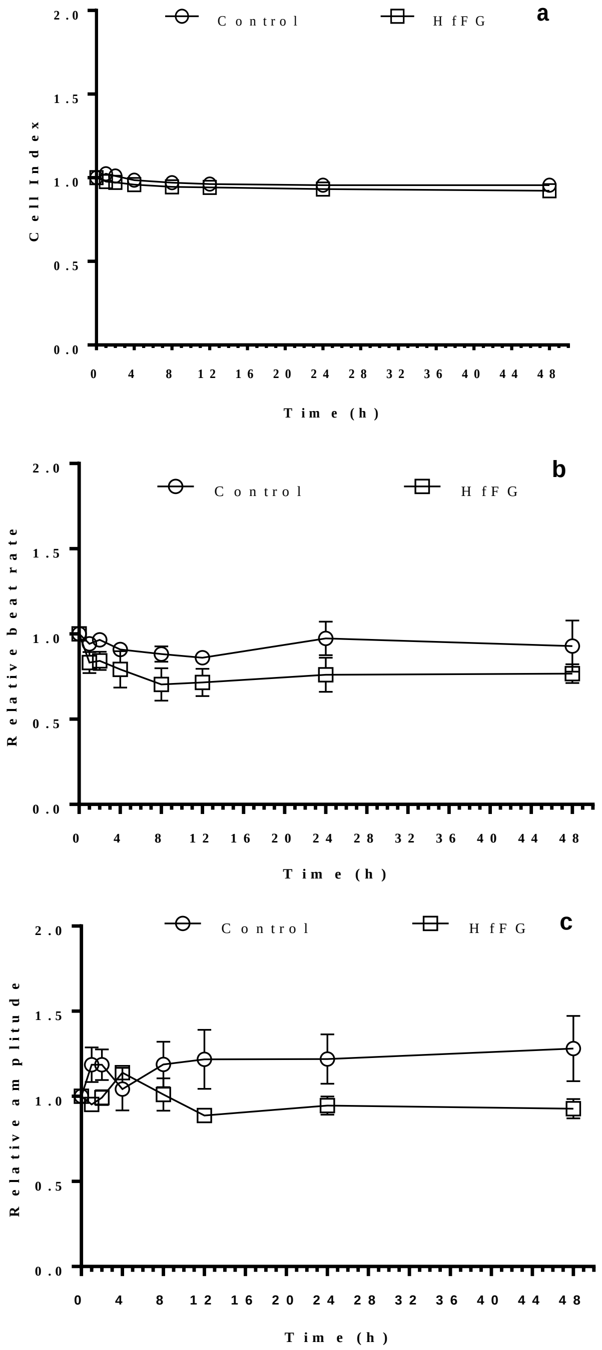

Fig. S6 Effects of HfFG on the function of human myocardial cells

Effects of HfFG on the proliferation (a), beat rate (b) and contractility (c) of human myocardial cells. Experiments were conducted in serum culture medium, HfFG was at 20 μg/mL, each predose parameter was normalized to 1, then the cell index, relative beat rate or relative amplitude in each group were calculated, Mean ± SEM, n = 4.
